# Progress of Section “Biochemistry” in 2022

**DOI:** 10.3390/ijms24065873

**Published:** 2023-03-20

**Authors:** Claudiu T. Supuran

**Affiliations:** Pharmaceutical and Nutraceutical Section, Department of NEUROFARBA, University of Florence, Via U. Schiff 6, 50019 Sesto Fiorentino, Firenze, Italy; claudiu.supuran@unifi.it

## 1. State of the Art

Of more than 16,400 papers published in 2022 in *International Journal of Molecular Sciences* [1], around 30–35% appeared in the Biochemistry section, which is a clear sign of the dynamic nature of this research field. The huge number of high-quality publications in all fields connected to Biochemistry and related areas (chemical biology, medicinal chemistry, molecular biology, etc.) undoubtedly demonstrates that the journal achieved a well-deserved maturity and visibility, as groups from all over the world contributed with relevant research in various topics over the last year, publishing original articles and reviews of considerable interest. Considering thus the significant number of papers which were published, I will only highlight some of the relevant developments in the main research areas which I consider of relevance.

Enzymes continue to be highly investigated proteins due to their involvement in all life processes in organisms all over the phylogenetic tree. More than 4000 papers dealing with these proteins were published in 2022 in the journal. I will highlight some of the research on one of my favorite proteins, the carbonic anhydrases (CAs, EC 4.2.1.1), which catalyze the reversible conversion between CO_2_ and bicarbonate [2]. As such, these enzymes distributed all over the phylogenetic tree, in prokaryotes and eukaryotes, play fundamental physiological roles but are also involved in many pathologies [3]. Furthermore, their environmental roles in processes connected to photosynthesis, global warming and sea acidification started to be understood only recently [4,5,6].

A relevant number of papers dealing with this enzyme, its inhibitors, activators and involvement in various diseases have been published in 2022 in this journal [7,8,9,10,11,12,13,14,15,16,17,18,19,20,21,22,23,24,25,26,27,28,29]. The first group of contributed materials dealt with the use of this protein for investigations of basic biochemical approaches, such as protein folding [7], thermodynamic parameters assessment for protein–ligand interactions [8], bioluminescence resonance energy transfer connected to the binding of the metal ion to apoenzymes [9], the possibility to evidence chalcogen bonds in the X-ray crystal structures of CA–lig and adduct [10]. In two other papers, the role of CAs in CO_2_ sequestration processes for alleviating global warming [11] or in photosynthesis in bacteria and plants (only the α-class CAs were considered in a very informative review by De Simone’s group [12]) were thoroughly discussed, proving again the potential environmental applications of these enzymes.

A large number of CA-related papers dealt with the drug design of CA inhibitors (CAIs) with various applications as anticancer agents (both for treatment and imaging) [13,14,15,16,17,18,19], antineuropathic pain compounds [20], mountain sickness leads [21], antiglaucoma agents [22] or antibacterials with a novel mechanism of action which, unlike classical antibiotics, target bacterial CAs from various pathogens [23,24,25]. Both sulfonamide and non-sulfonamide compounds have been reported in these interesting papers, which highly enrich the number of such pharmacological agents useful for the management of a multitude of pathological conditions [2,3].

The role of the genetic deficiency of the mitochondrial enzyme CA VA which causes hyperammonemia in the affected patients has also been investigated [26] as well as the role of immune checkpoint blockade on the anticancer effects of CA IX chimeric antigen receptor T cells [27]. A comprehensive review on CAs present in the model organism zebra fish has also been published by Parkkila’s group [28]. An interesting work from Pastorek’s group revealed that CA IX, usually connected with hypoxic tumors, is also present in abdominal aortic aneurysms tissue and plasma [29].

A consistent number of papers published in 2022 deal with the understanding of tumorigenesis and the development of anticancer agents, with more than 3800 such contributions found. Again, I will highlight only those who seemed more innovative and promising to me. Thus, Nakamura et al. [30] investigated ribosomal stress and hypoxia in oral cavity tumors, whereas Ferreira et al. [31] explored the role of targeting lysosomes in colorectal cancer. Campagna et al. [32] reported an interesting study regarding the role of paraoxonase-2, an enzyme with a debated role in tumorigenesis, in oral squamous cell carcinomas, whereas in another interesting review article, the possibility to target the isoprenoid biosynthetic pathway in multiple myeloma is presented in detail [33]. An overview of metabolism and signal transduction in cancer cells was presented by Chae and Hong [34], who stressed the fact that metabolism of cancer cells is thoroughly relevant both for the design of novel therapies and for the outcome of the existing ones [35,36]. Finally, targeting glutamine [37] in cancer metabolism became an interesting new approach which has been also investigated by Kao et al. [38]. Together with ferroptosis [39], this represents one of the most relevant antitumor mechanisms ultimately explored.

More than 1350 papers were published in 2022 on anti-infectives. Among them, I will highlight an interesting review on photodynamic therapy for the inactivation of *Acinetobacter baumannii* biofilms [40], a computational approach for designing anti-*Staphylococcus aureus* agents by targeting transcription [41], antimicrobial peptides showing broad-spectrum activity against *Pseudomonas aeruginosa* [42], silver nanoparticles for delivery of antibiotics against *Escherichia coli* infections [43], as well as a comprehensive review regarding the nanoparticles for antibacterial delivery [44].

The antiviral landscape was obviously dominated by SARS-CoV-2 studies. Interesting SARS-CoV-2 main protease inhibitors were reported by Geiger et al. [45], whereas Ma et al. [46] identified darunavir derivatives which effectively inhibit the cysteine-like protease of this virus. Many studies on monoclonal antibodies targeting various proteins of the virus were also ultimately published [47,48,49,50,51], although the role of these therapeutics in the management of COVID-19 was very much re-dimensioned due to mutations and loss of efficacy as well as the design of small molecule inhibitors targeting various viral proteins [52,53]. An interesting review on the other virus which worried the world in the last year, monkeypox, has also been timely published in the journal [54].

## 2. Future Prospects

The Biochemistry section of the journal receives a large number of submissions and will continue the publication of high-quality research. Given the interdisciplinary nature of the journal, it is sometimes difficult to classify a submission as being mainly biochemical, pharmacological or strictly a medical work; this is, of course, a wealth for the journal, but also a source of potential problems, as it is not always easy to invite the best reviewers for such interdisciplinary submissions. Furthermore, this problem of finding appropriate reviewers is a general phenomenon in all peer review processes, which is, however, complicated in this journal due to specific requirements which should be simplified.

The increase in the impact factor of the journal over the last years, the large number of citations of the published papers, and the number of submissions per se demonstrate that the journal is appreciated by the scientific community worldwide, but as editors, we should continue to improve the workflow and the quality of the published papers.

## Data Availability

Not applicable.
